# Protein interactors of 3-*O* sulfated heparan sulfates in human MCI and age-matched control cerebrospinal fluid

**DOI:** 10.1038/s41597-023-02009-1

**Published:** 2023-03-06

**Authors:** Andreia Ferreira, Evy Timmerman, An Staes, Marnik Vuylsteke, Louis De Muynck, Kris Gevaert

**Affiliations:** 1 Janssen Research & Development, a Division of Janssen Pharmaceutica N.V., 2340 Beerse, Belgium; 2 VIB-UGent Center for Medical Biotechnology, B-9052 Ghent, Belgium; Department of Biomolecular Medicine, Ghent University, B-9052 Ghent, Belgium; 3 VIB Proteomics Core, B-9000 Ghent, Belgium; Department of Biomolecular Medicine, Ghent University, B-9000 Ghent, Belgium; 4 Gnomixx, B-9090 Melle, Belgium

**Keywords:** Proteomic analysis, Cellular neuroscience

## Abstract

Heparan sulfates (HS) proteoglycans are commonly found on the cell surface and mediate many processes. Binding of HS ligands is determined by the sulfation code on the HS chain that can be *N*-/2-*O*/6-*O*- or 3-*O*-sulfated, generating heterogenous sulfation patterns. 3-*O* sulfated HS (3S-HS) play a role in several (patho)physiological processes such as blood coagulation, viral pathogenesis and binding and internalization of tau in Alzheimer’s disease. However, few 3S-HS-specific interactors are known. Thus, our insight into the role of 3S-HS in health and disease is limited, especially in the central nervous system. Using human CSF, we determined the interactome of synthetic HS with defined sulfation patterns. Our affinity-enrichment mass spectrometry studies expand the repertoire of proteins that may interact with (3S-)HS. Validating our approach, ATIII, a known 3S-HS interactor, was found to require GlcA-GlcNS6S3S for binding, similar to what has been reported. Our dataset holds novel, potential HS and 3S-HS protein ligands, that can be explored in future studies focusing on molecular mechanisms that depend on 3S-HS in (patho)physiological conditions.

## Background & Summary

Heparan sulfates (HS) are glycosaminoglycans covalently attached to proteins that are mainly found at the cell membrane or in the extracellular matrix^1^. HS are linear polysaccharide structures made from repeating disaccharide building blocks consisting of (N-acetylated/sulfated) glucosamine (GlcN) and a hexuronic acid (glucuronic acid (GlcA) or iduronic acid (IdoA)). The last step in the biosynthesis of HS is carried out by different HS sulfotransferases which place sulfate groups at different positions of the disaccharide unit, resulting in *N*-/2-*O*/6-*O*- and 3-*O*-sulfated HS chains. In this way, highly heterogeneous and unique sulfation patterns are created that mediate interactions of Heparan Sulfates Proteoglycans (HSPGs) with their ligands^2^.

HSPGs interact with a plethora of ligands involved in various physiological processes such as cell proliferation, cell signaling, tissue distribution of molecules such as growth factors and morphogens, immunity and inflammation^3^. Several HS interactors have been described, contributing to our knowledge of the physiological relevance of HSPGs. Few reports have however focused on profiling proteins that preferentially bind to 3-*O* sulfated HS (3S-HS) and only a limited number of 3S-HS ligands are known. Antithrombin III (ATIII)^4^, herpes simplex virus-1 (HSV-1) glycoprotein D (gD)^5^, the spike glycoprotein of SARS-CoV2^6^, neuropilin-1 (NRP1)^7^, cyclophilin B^8^, stabilin^9^, fibroblast growth factors receptors (FGFRs) and FGFs ligands^10,11^ were reported to show preferential binding to 3S-HS. Such studies have highlighted the roles of 3S-HS in blood coagulation, viral pathogenesis, and, more recently, synaptogenesis. The NRP1 protein was first identified as an *Hs3st1*-modified HS interactor in human sera^7^, and 3-*O* HS sulfation was reported to promote NRP1 binding to HS, resulting in an inhibition of the growth cone collapse in murine primary neurons *in vitro*. A recent study identified 3S-HS ligands associated with neuronal development and suggested a role in synaptogenesis and neuronal excitability^12^. These studies, in rodents, bring forward a pivotal role for 3S-HS in neuronal development however, the role of 3S-HS in the adult human brain remains unknown.

The HS 3-*O*-sulfotransferases (HS3STs) modify the C-3 position of the GlcN residue and the HS3ST family has seven isoforms. Little is known about their (patho-)physiological role. A growing body of evidence highlights a putative contribution of HSPGs^13^ and, more specifically, HS 3-*O* sulfation in several pathological processes, one being the propagation of the tau protein in Alzheimer’s disease (AD). Four of the seven HS3ST isoforms are predominantly expressed in AD-affected brain regions^14,15^. One study has shown increased levels of *HS3ST2* and *HS3ST4* mRNA in the hippocampus of AD patients and reported HS3ST2 to be critical for abnormal phosphorylation of the tau protein^16^. Further, recent GWAS studies identified several SNPs in the *HS3ST1* and *HS3ST5* gene loci associated with AD^17,18^. Moreover, studies have brought forward a contribution of 3-*O* sulfation to the internalization and binding of monomeric^19^ and aggregated^20^ tau. Given the potential contribution of 3S-HS to pathology in neurodegenerative disorders such as AD, understanding the mechanisms, pathways and ligands dependent on 3S-HS is relevant to understand disease etiology.

In this work, we identified the proteins binding preferentially to HS and HS carrying 3-*O* sulfate groups (3S-HS) using cerebrospinal fluid (CSF) from mild cognitive impaired (MCI) patients and age-matched controls. CSF was here used as a surrogate for the extracellular environment of the central nervous system. To study these HS and 3S-HS interactomes, affinity-enrichment-mass spectrometry (AE-MS)^21^ was used to detect CSF proteins bound to one of four different synthetic HS: two different HS carrying 3-O sulfation and two different HS without 3-*O* sulfation. Proteins of which HS binding was dependent on 3-*O* sulfation were identified. ATIII was identified as a 3S-HS specific interactor^4^, validating our strategy. In summary, we provide a resource valuable for future studies on molecular mechanisms that depend on HS (3-*O*) sulfation.

## Methods

### Cerebrospinal Fluid sample information

**Fig. 1:**
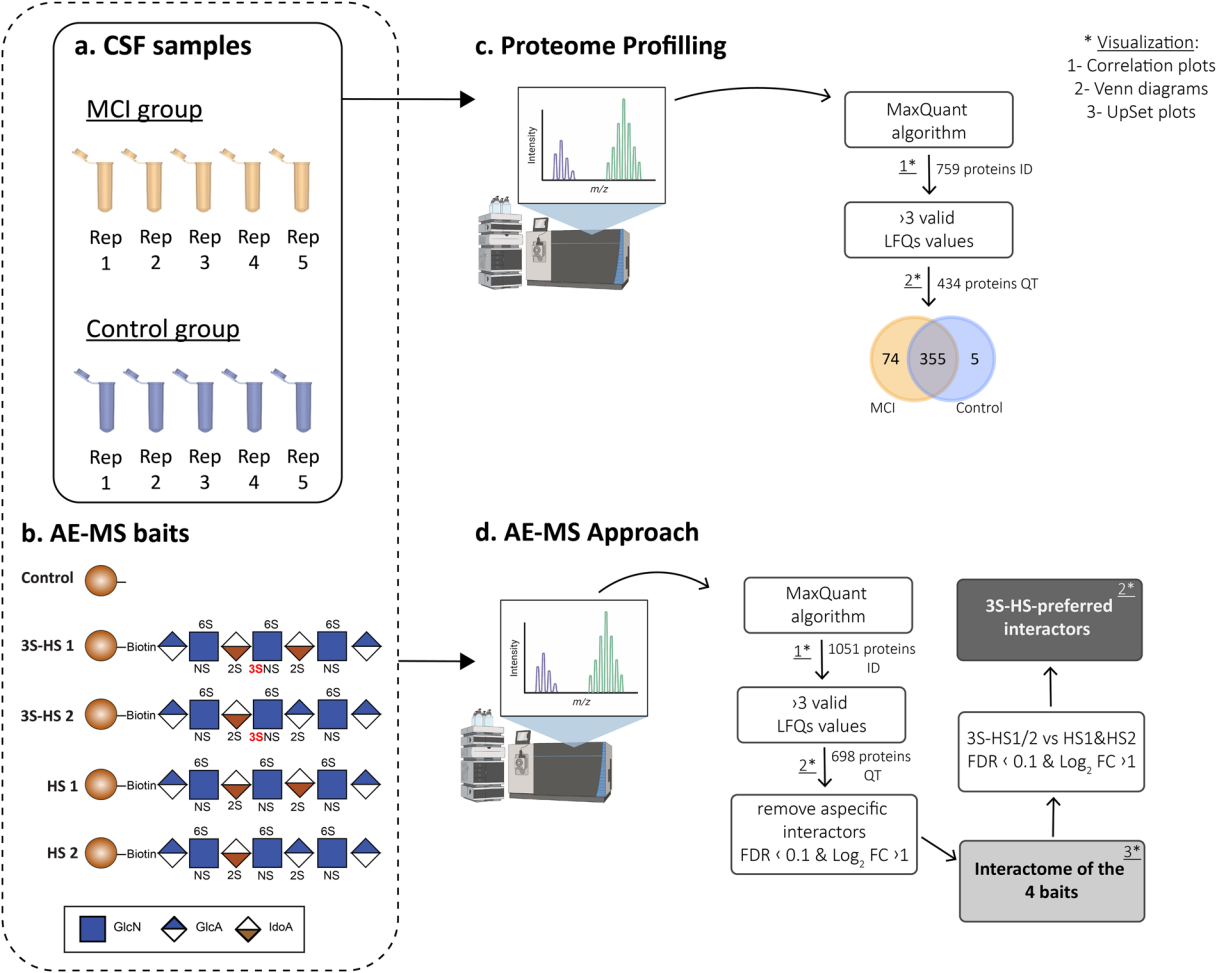
Experimental and analysis workflow. (**a**) 5 CSF samples from different individuals were used for each sample group (MCI and control) as inputs for the proteome profiling and affinity-enrichment (AE)-MS. (**b**) 5 different conditions were used for the AE-MS approach: (1) control with beads-only; (2) 3S-HS1; (3) 3S-HS 2; (4) HS 1; (5) HS 2. (**c**) LFQ proteomics was used to profile the proteomes of the 10 CSF samples. The LC-MS/MS data were analyzed by MaxQuant and, from the 759 proteins identified (ID), only those with a minimum of 3 LFQ valid values in at least one group were withheld, resulting in 434 reliably quantified (QT) proteins. Of these proteins, 74 proteins were found only in the MCI samples, 5 only in the control samples, and 355 were shared between both types of samples. When comparing the proteomes, no proteins were found significantly regulated in either the MCI or the control sample groups. (**d**) AE-MS was used to profile the interactomes of the synthetic HS baits in MCI and control samples. To identify non-specific binders, a beads-only control condition was used. The LC-MS/MS data were identified by MaxQuant, which resulted in the identification of 1,051 proteins. A further selection of proteins with a minimum of 3 valid LFQ values in at least one condition resulted in 698 reliably quantified proteins. To profile the interactomes, proteins with equal or higher levels of expression in the beads-only condition were excluded. Next, proteins upregulated in the 3S-HS 1 or 3S-HS 2 conditions compared to HS 1 and HS 2 conditions were selected as interactors showing preferential binding to HS containing 3-O sulfation (3S-HS interactors). Finally, 3S-HS interactors were compared between the MCI and Control sample groups, but no proteins were found at significantly different levels.

Cerebrospinal Fluid (CSF) samples of five mild cognitive impaired (MCI) patients and five age-matched controls (experimental overview in Fig. 1) were collected according to the recommended protocol for CSF collection and biobanking^22^. The samples were obtained from the Janssen Biobank, provided by Dr. Katrin Haeverans. The study from which the samples originate (ClinicalTrials.gov Identifier: NCT03375697) was approved by the Ethics Committee of the University of Antwerp Hospital from Belgium, the METC of the BEBO foundation from the Netherlands and the CEIC Hospital Clínico San Carlos de Madrid from Spain. The participants (or a legally acceptable representative) provided their written informed consent for research. The main clinical features of the patients are summarized in Table 1.

**Table 1:** Subjects’ main clinical features and CSF biomarkers.

Subject	Group	Replica	Gender	Age	CDR score	Aβ_42_ (ng/L)	Aβ_42_ /Aβ_40_ (ng/L)	p181 tau (ng/L)	Protein (mg/ml)
1	MCI	1	Male	67	1	361	0.0396	112	1.02
2	MCI	2	Female	72	0.5	285	0.0434	130	0.94
3	MCI	3	Male	72	1	418	0.0438	76	1.38
4	MCI	4	Male	74	0.5	323	0.0470	79	0.86
5	MCI	5	Female	74	1	491	0.0430	70	0.74
6	Control	1	Female	71	—	657	0.0869	39	0.90
7	Control	2	Male	67	—	655	0.101	43	1.04
8	Control	3	Female	75	—	326	0.0379	64	1.02
9	Control	4	Male	70	—	642	0.0866	48	1.47
10	Control	5	Male	75	—	561	0.0911	47	0.96

**CDR score:** The CDR (Clinical Dementia Rating Scale) is a global clinical scale with established diagnostic and severity-ranking utility widely used in clinical trials. The CDR assesses three domains of cognition (memory, orientation, judgment/problem solving) and three domains of function (community affairs, home/hobbies, personal care) using structured interviews of both the study subject and a companion/informant. CDR score ranges from 0 to 3 with 0 having no dementia and 3 having severe dementia.

### Sample preparation for proteome analysis

To profile the CSF proteomes, 100 µg of protein material was taken from each sample to which 100 µl of 100 mM HEPES (pH 7.5) was added. Proteins were reduced and alkylated by adding TCEP-HCl and iodoacetamide (IAA) to final concentrations of 15 mM and 30 mM respectively, and samples were incubated for 15 min at 37 °C in the dark. Next, proteins were digested by first adding 1 µg of endoproteinase-LysC (Wako) and incubating the samples for 3 h at 37 °C, followed by the addition of 1 µg of sequencing grade modified trypsin (Promega) and incubation of the samples overnight at 37 °C. To stop the digestion, 10% TFA was added until a pH of 2-3 was reached. The peptide mixtures were then purified using OMIX C18 tips (Agilent) according to the manufacturer’s instructions. Peptides were vacuum-dried completely using a SpeedVac (Thermo Fischer) and stored at −20 °C until further use.

### LC-MS/MS analysis for proteome profiling

Prior to analysis, each peptide sample was solubilized in 20 µL loading solvent A (0.1% TFA in water/acetonitrile (ACN) (98/2, v:v)). The peptide concentration was measured using a Dropsense16 (Unchained Labs) and per sample, 2 µg was injected for LC-MS/MS analysis using an Ultimate 3000 RSLCnano system in-line connected to an Orbitrap Fusion Lumos mass spectrometer (Thermo Fisher). Peptide trapping was performed at 10 μl/min for 4 min in loading solvent A on a 20 mm trapping column (made in-house, 100 μm internal diameter (I.D.), 5 μm beads, C18 Reprosil-HD, Dr. Maisch, Germany). The peptides were separated on a 200 cm µPAC™ column (C18-end-capped functionality, 300 µm wide channels, 5 µm porous-shell pillars, inter pillar distance of 2.5 µm and a depth of 20 µm; PharmaFluidics) that was kept at a constant temperature of 50 °C. Peptides were eluted using a linear gradient reaching 55% solvent B (0.1% formic acid (FA) in water/ACN (20/80, v/v)) after 115 min and 99% MS solvent B at 120 min, followed by a 10-min wash at 99% solvent B and re-equilibration with solvent A’ (0.1% FA). During the first 15 min, the flow rate was set to 750 nl/min, after which it was kept constant at 300 nl/min.

The mass spectrometer was operated in data-dependent mode, automatically switching between MS and MS/MS acquisition in TopSpeed mode. Full-scan MS spectra (300–1,500 m/z) were acquired at a resolution of 120,000 in the Orbitrap analyzer after accumulation to a target AGC value of 200,000 with a maximum injection time of 250 ms. The precursor ions were filtered for charge states (2–7 required), dynamic exclusion (60 s; ± 10 ppm window) and intensity (minimal intensity of 3E4). The precursor ions were selected in the ion routing multipole with an isolation window of 1.2 Da and accumulated to an AGC target of 5E3 or a maximum injection time of 40 ms and activated using HCD fragmentation (34% NCE). Peptide fragments were analyzed in the Ion Trap analyzer at a normal scan rate.

### Data analysis for proteome profiling

Analysis of the mass spectrometry data was performed in MaxQuant (version 1.6.17.0) with mainly default search settings including a false discovery rate set at 1% on peptide-to-spectrum matches (PSMs), peptide and protein levels. Spectra were searched against the human protein sequences stored in the Swiss-Prot database (UPhuman_9606, database release version of January 2021, containing 20,394 protein sequences, downloaded from http://www.uniprot.org). The mass tolerance for precursor and fragment ions was set to 4.5 and 20 ppm, respectively, during the main search. Enzyme specificity was set as C-terminal to arginine and lysine, also allowing cleavage at arginine- and lysine-proline bonds with a maximum of two missed cleavages. Variable modifications were set to oxidation of methionine residues and acetylation of protein N-termini. Carbamidomethylation of cysteine residues was set as a fixed modification. Matching between runs was enabled with a matching time window of 0.7 min and an alignment time window of 20 min. Only proteins with at least one unique or razor peptide were retained.

Proteins were quantified by the MaxLFQ algorithm integrated into the MaxQuant software. A minimum ratio count of two unique or razor peptides was required for quantification. A total of 53,296 PSMs were identified, resulting in 4,054 identified unique peptides corresponding to 759 proteins, of which 434 protein groups could be reliably quantified (i.e., protein groups with at least 3 valid LFQ intensity values in one of the experimental conditions). The full list of proteins quantified in the affinity-enrichment assay, with the corresponding Log_2_ fold change, p-value, FDR and number of proteins where they were identified can be found in the Figshare Repository (Proteins List^23^).

**Fig. 2:**
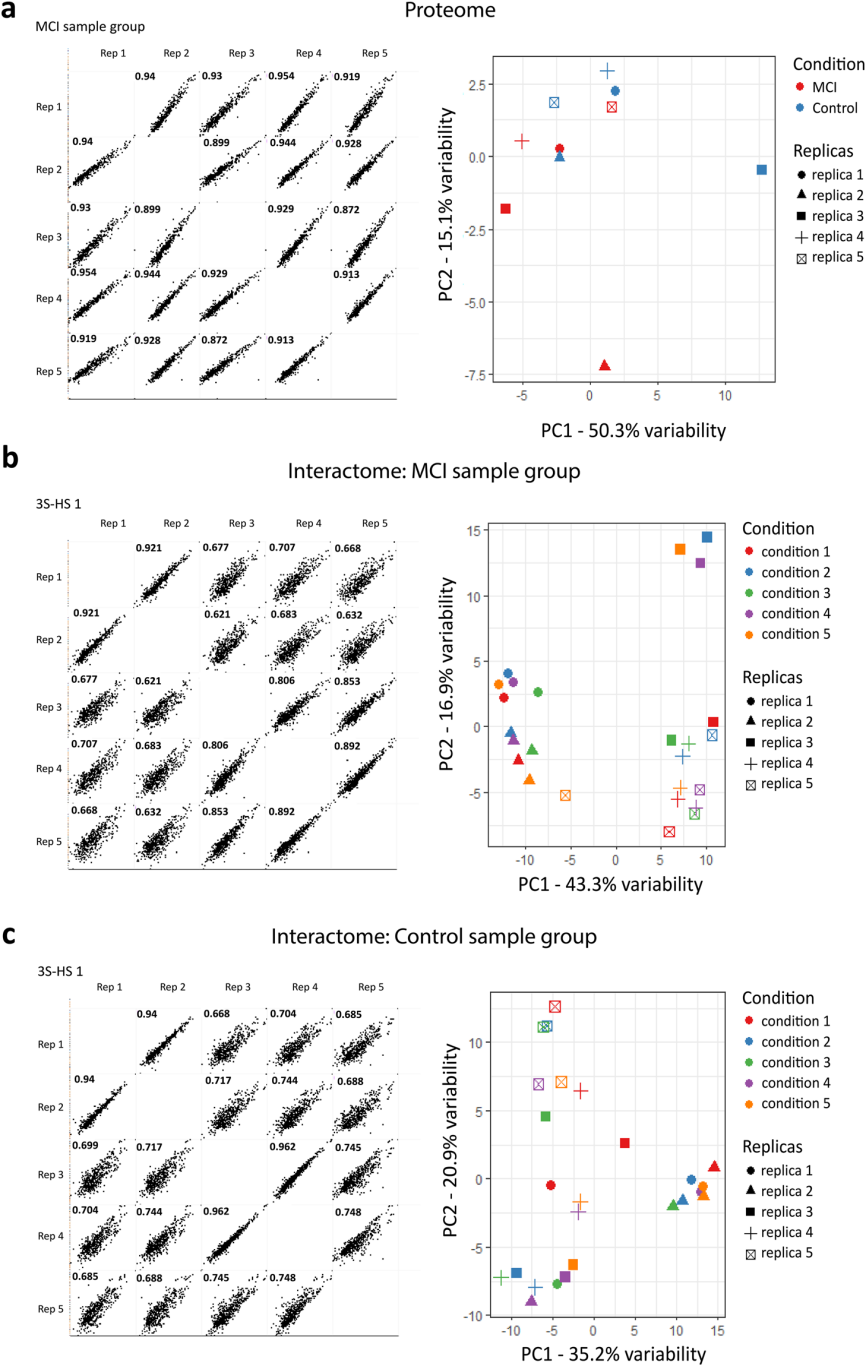
Assessment of data variability between replicas. To assess the variability of the different CSF samples, of the (**a**) proteome and interactomes of the (**b**) MCI and the (**c**) control sample groups, correlation plots (left panels) using identified proteins as variables to compare the different replicas were performed. PCA analyses (right panels) were performed on the replicate samples using all quantified proteins as variables. The remaining profile plots are available in the Figshare repository (Correlation Plots^23^).

Data analysis was performed with the Perseus software (version 1.6.2.1) after loading the *proteingroups* file from MaxQuant. Reverse database hits and contaminants were removed, and LFQ intensity values were log_2_-transformed. The data were imputed to replace missing values with random, low numbers that are drawn from a normal distribution. Missing values reflect low abundance measurements; hence the default parameters were used as they mimic this case. To assess the data variability between replicas within each sample group, correlation plots with all LFQ values of the identified proteins were generated (Fig. 2a left panel; Correlation Plots^23^). Here, only the profile plot of the MCI sample group is shown, and all other profile plots are available in the Figshare Repository (Correlation Plots^23^). In addition, a principal component analysis (PCA) was performed on the replicate samples using all quantified proteins as variables (Fig. 2a right panel). To compare protein intensities in both sample sets, statistical testing for differences between the means of the two sample groups was performed using the package limma^24^.

All proteomics data have been deposited to the ProteomeXchange Consortium via the PRIDE partner repository^25^. Annotations linking the raw data files to the experimental conditions can be found in Table 5.

### Affinity-enrichment studies

To profile the interactome of 3-*O* sulfated heparan sulfates (3S-HS), four different synthetic biotinylated HS (kindly provided by Prof. Jian Liu, Glycan Therapeutics) were used as baits to pull-down their interacting proteins from the CSF samples. Two 3S-HS structures were used as baits; one contains an HSV-1 gD-like binding site (3S-HS 1) and the other one an ATIII-like binding site (3S-HS 2). The remaining two HS had the same sulfation pattern of 3S-HS 1 and 3S-HS 2 (HS 1 and HS 2, respectively) but without the 3-*O* sulfation in the glucosamine residue and were used as controls for identifying preferential interactors of the 3S-HS. The structure of the four synthetic HS is shown in Fig. 1b. Pull-downs were performed using Streptavidin Mag Sepharose (GE Healthcare) magnetic beads, following the manufacturer’s protocol. Briefly, for the beads-HS conjugation, 20 µl of beads slurry was washed with 500 µl of 50 mM Tris-HCl (Invitrogen), 150 mM NaCl (Invitrogen), pH 7.5 (TBS) buffer and incubated with 300 µl of 0.16 mg/ml of synthetic HS or PBS (AE control), for 90 min at 4 °C and 30 min at room temperature (RT), whilst shaking with a HulaMixer (Invitrogen). The beads were thoroughly washed with 500 µl TBS and incubated with 200 µl of CSF overnight at 4 °C in a HulaMixer. The beads were again thoroughly washed, initially with 500 µl TBS and next with 500 µl 20 mM Tris-HCl pH 8.0 (Invitrogen), 2 mM CaCl_2_ (Sigma-Aldrich) (trypsin digestion buffer). The beads were finally resuspended in 150 µl trypsin digestion buffer. This protocol was repeated using the four different synthetic HS and for the CSF samples from the 10 different individuals.

To identify the interacting proteins, an on-beads digestion protocol was used. Briefly, 5 µl of 0.2 µg/µl of sequencing grade modified trypsin (Promega) was added to the beads in trypsin buffer. Samples were incubated for 4 h at 37 °C, whilst shaking at 1,200 rpm, followed by a centrifugation step for 15 min at 15,000 x*g* and RT. The supernatant was transferred to a new Eppendorf tube and 5 µl of 0.2 µg/µl of trypsin was added. The solution was incubated overnight at 37 °C, whilst shaking at 750 rpm. To extract and purify the peptides, OMIX C18 tips (Agilent) were used as described before, following which peptides were vacuum-dried.

### LC-MS/MS analysis of the heparan sulfates interactors

Peptides were re-dissolved in 20 µl loading solvent A (0.1% TFA in water/ACN (98/2; v/v)) of which 5 µl was injected for LC-MS/MS analysis on an Ultimate 3000 RSLC nano-LC (Thermo Fisher) in-line connected to a Q Exactive mass spectrometer (Thermo Fisher). The sample mixture was first loaded on a trapping column (made in-house, 100 μm I.D. × 20 mm, 5 μm beads C18 Reprosil-HD, Dr. Maisch). After flushing from the trapping column, the peptides were separated on a 50 cm µPAC™ column with C18-end-capped functionality (PharmaFluidics) kept at a constant temperature of 35 °C. Peptides were eluted by a stepped gradient from 98% solvent A’ (0.1% FA) to 30% solvent B’ (0.1% FA in water/acetonitrile, 20/80 (v/v)) in 75 min up to 50% solvent B’ in 15 min, followed by a 5 min wash reaching 95% solvent B’, all at a stepped flow rate starting from 750 nl/min for 9 min to 300 nL/min until the end of the run.

The mass spectrometer was operated in data-dependent, positive ionization mode, automatically switching between MS and MS/MS acquisition for the 5 most abundant peaks in a given MS spectrum. The source voltage was 3 kV and the capillary temperature was 275 °C. One MS1 scan (m/z 400−2,000, AGC target 3 × E6 ions, maximum ion injection time 80 ms), acquired at a resolution of 70,000 (at 200 m/z), was followed by up to 5 tandem MS scans (resolution 17,500 at 200 m/z) of the most intense ions fulfilling predefined selection criteria (AGC target 50,000 ions, maximum ion injection time 80 ms, isolation window 2 Da, fixed first mass 140 m/z, spectrum data type: centroid, intensity threshold 1.3E4, exclusion of unassigned, 1, 5–8, >8 positively charged precursors, peptide match preferred, exclude isotopes on, dynamic exclusion time 12 s). The HCD collision energy was set to 25% NCE and the polydimethylcyclosiloxane background ion at 445.120025 Da was used for internal calibration (lock mass). QCloud^26^ was used to control instrument longitudinal performance during the project.

### Analysis of the interactome profiling data

MS/MS data analysis for the identification and quantification of proteins was performed as described above (section Data analysis for the proteome profiling). Here, in all 25 samples of the interactomes isolated from the control group, 158,803 PSMs, 4,899 peptides, and 827 protein groups were identified. Of those, 569 proteins were considered reliably quantified, i.e., proteins with at least 3 valid LFQ intensity values in one of the experimental conditions. In all 25 samples of the interactomes isolated from the MCI sample group, 168,765 PSMs, 5,574 peptides, and 936 protein groups were identified. And, of those, 590 proteins were considered reliably quantified. A total of 698 proteins were quantified between MCI and controls groups. The full list of proteins quantified in the affinity-enrichment assay, with the corresponding Log_2_ fold change, p-value, FDR and number of proteins where they were identified can be found in the Figshare Repository (Proteins List^23^).

Data analysis was performed with the Perseus software (version 1.6.2.1) following the same procedure as done previously for the proteome data analysis. Similarly, to assess the data variability, between replicas within each sample group, correlation plots with all identified proteins as variables were performed (Fig. 2 left panel; Correlation Plots^23^). Figure 2b,c show representative correlation plots of the 3S-HS 1 condition for the sake of visualization (profile plots of the other conditions can be found in the Figshare repository^23^). To evaluate the variability of the samples, PCA was performed on the replicate samples using all quantified proteins as variables (Fig. 2 right panel).

Statistical analyses were performed with the combined datasets of the MCI and control sample groups. For this, the experiment was laid out as a complete block design with five replicates per disease group, having each of the five conditions within each of the five replicates. Each of the 698 proteins was analyzed separately by fitting a linear mixed model of the following form (random terms underlined): response = μ + treatment + replicates(sample group) + error. The response represents the log_2_-transformed LFQ intensity measured, the treatment represents the 10 combinations of the two sample group states (MCI and control) and five AE conditions (control, 3S-HS1, 3S-HS2, HS1, and HS2), replicates(sample group) represents the five replicas nested into one sample group, and the error term represents the random noise. Linear mixed models were fitted by the residual maximum likelihood (REML) approach as implemented in Genstat v21 software^27^. The significance of treatment effects was assessed by an approximate F-test as implemented in Genstat v21^27^. The significance of individual comparisons between the levels of the treatment factor was assessed by a t-test. Correction for multiple testing was done by estimating the false discovery rate (FDR) by modeling significance values as a 2-component mixture of Uniform and Beta or Gamma densities^28^ as implemented in Genstat v21^27^.

**Fig. 3:**
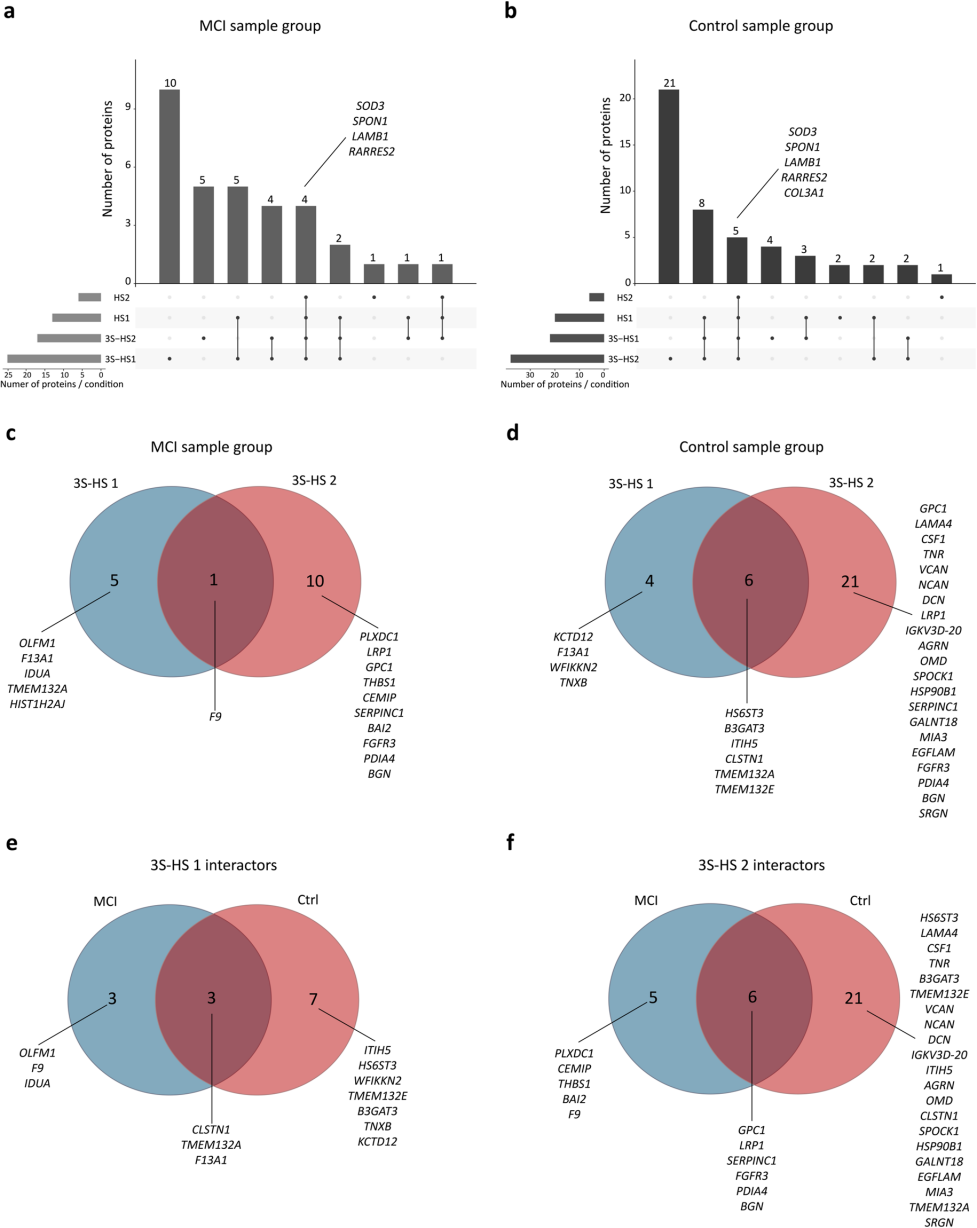
Interactions of the different interactomes. (**a**,**b**) UpSet plots were used to visualize the proteins intersection between the different interactomes for both the (**a**) MCI and (**b**) Control sample groups. Proteins shared between the 4 HS are listed. (**c**,**d**) Venn diagrams showing shared and unique 3S-HS-preferred interactor proteins between 3S-HS 1 and 3S-HS 2 conditions for both the (**c**) MCI and the (**d**) Control sample groups. (**e**,**f**) Venn diagrams visualizing the (**e**) 3S-HS 1 and (**f**) 3S-HS 2 interactors shared between the MCI and the Control sample groups. All proteins belonging to each intersection are listed.

The pairwise comparisons performed in the analysis described before are demonstrated in Table 2 (for MCI sample group) and Table 3 (for control sample groups), with the corresponding number of proteins selected. This analysis allowed the interactome profiling of the four baits (comparisons 1, 2, 3, 4, 7, 8, 9 and 10), and the proteins enriched in the 3S-HS conditions (comparisons 5, 6, 11 and 12). An overview of the list of interactomes of the different conditions and their p-value and FDR values are available in the Figshare Repository (Proteins List^23^). UpSet plots^29^ were used to visualize the intersection between the interactomes of the four conditions of each sample group (Fig. 3a,b).

**Table 2:** List of pairwise comparisons in the MCI sample group.

Comparison	Resulting list	Pairwise comparison (AE conditions)	Number of proteins selected
1	3S-HS 1 interactome	3S-HS 1 vs control	24
2	3S-HS 2 interactome	3S-HS 2 vs control	17
3	HS 1 interactome	HS 1 vs control	14
4	HS 2 interactome	HS 2 vs control	6
5	Proteins enriched in 3S-HS 1	3S-HS 1 vs HS 1 & 3S-HS 1 vs HS 2	6
6	Proteins enriched in 3S-HS 2	3S-HS 2 vs HS 1 & 3S-HS 2 vs HS 2	11

**Threshold for protein selection**: log_2_ LFQs intensity significantly increased (FDR < 0.1, log_2_ FC > 1).

**Table 3:** List of pairwise comparisons in the control sample group.

Comparison	Resulting list	Pairwise comparison (AE conditions)	Number of proteins selected
7	3S-HS 1 interactome	3S-HS 1 vs control	22
8	3S-HS 2 interactome	3S-HS 2 vs control	38
9	HS 1 interactome	HS 1 vs control	20
10	HS 2 interactome	HS 2 vs control	6
11	Proteins enriched in 3S-HS 1	3S-HS 1 vs HS 1 & 3S-HS 1 vs HS 2	10
12	Proteins enriched in 3S-HS 2	3S-HS 2 vs HS 1 & 3S-HS 2 vs HS 2	27

**Threshold for protein selection**: log_2_ LFQs intensity significantly increased (FDR < 0.1, log_2_ FC > 1).

To profile the shared or unique 3S-HS interactors between MCI and control sample groups, pairwise comparisons were performed as described in Table 4, with the corresponding number of selected proteins. Venn diagrams were made (using the web tool Bioinformatics & Evolutionary Genomics from VIB-UGent - http://bioinformatics.psb.ugent.be/webtools/Venn/) to visualize unique or shared proteins between the 3S-HS 1 and 3S-HS 2 conditions, for both MCI (Fig. 3c) and control sample groups (Fig. 3d) with the corresponding list of proteins. Venn diagrams were also made to visualize unique or shared proteins between the MCI and control sample groups for both the 3S-HS 1 (Fig. 3e) or 3S-HS 2 (Fig. 3f) specific interactors, with the corresponding list of proteins.

**Table 4:** List of pairwise comparisons between MCI and control sample groups.

Comparison	Resulting list	Pairwise comparison (AE conditions)	Number of proteins selected
13	Proteins enriched in 3S-HS 1 different between Control and MCI	Comparison 11 vs Comparison 5	0
14	Proteins enriched in 3S-HS 2 different between Control and MCI	Comparison 12 vs Comparison 6	0

**Threshold for protein selection**: log_2_ LFQs intensity significantly different (FDR < 0.1, log_2_ FC > 1 (MCI)/−1 (control)).

**Table 5:** Proteome experiment.

Raw file name	Cohort
F004364_2p_200uPAC14_trap9_CMB-785-Andreia-CSF-170_9.raw	MCI
F004362_2p_200uPAC14_trap9_CMB-785-Andreia-CSF-184_8.raw	MCI
F004356_2p_200uPAC14_trap9_CMB-785-Andreia-CSF-162_6.raw	MCI
F004348_2p_200uPAC14_trap9_CMB-785-Andreia-CSF-248_2.raw	MCI
F004346_2p_200uPAC14_trap9_CMB-785-Andreia-CSF-151_1.raw	MCI
F004366_2p_200uPAC14_trap9_CMB-785-Andreia-CSF-232_10.raw	Control
F004358_2p_200uPAC14_trap9_CMB-785-Andreia-CSF-226_7.raw	Control
F004354_2p_200uPAC14_trap9_CMB-785-Andreia-CSF-244_5.raw	Control
F004350_2p_200uPAC14_trap9_CMB-785-Andreia-CSF-230_4.raw	Control
F004350_2p_200uPAC14_trap9_CMB-785-Andreia-CSF-216_3.raw	Control

Annotations of the raw data files and the corresponding cohort.

**Fig. 4:**
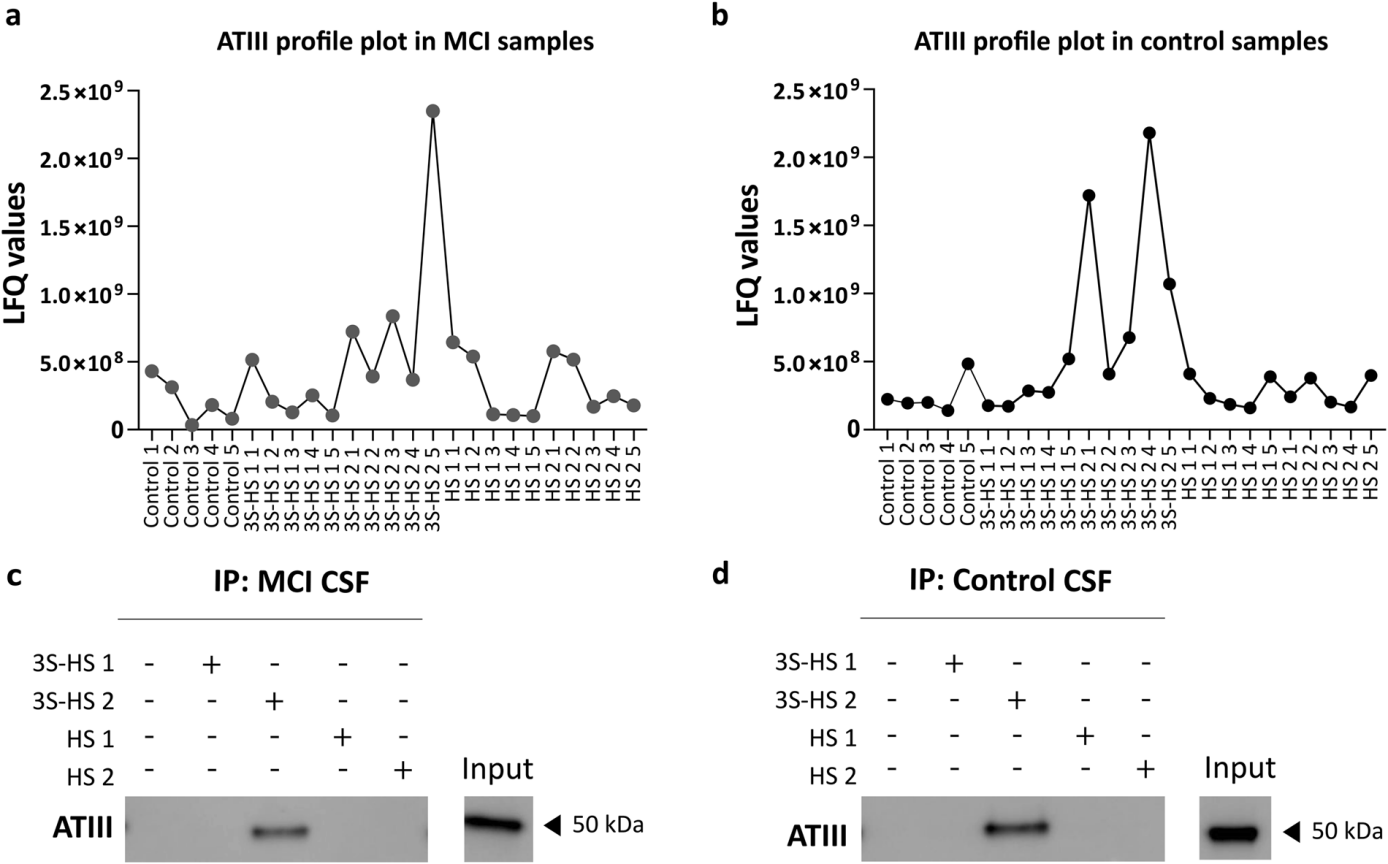
Binding profile of ATIII. The raw LFQ values for (**a**,**b**) ATIII were plotted for the different replicas of the different AE conditions. ATIII protein is more abundant in the 3S-HS 2 condition either in the (**a**) MCI or (**b**) control sample groups. To validate the dataset, the AE experiments were repeated with pooled CSF samples for each sample group. The presence of the ATIII protein in the eluted fraction was assessed via western blot. For both the (**c**) MCI and (**d**) control sample groups, ATIII presence was confirmed, but only in AE performed with 3S-HS 2 oligosaccharide. The input fraction corresponding to the pooled CSF samples is presented together with the molecular weight of each band observed.

To validate the dataset, the profile plot of ATIII (Fig. 4a), a known 3S-HS interactor, for each sample group between the different conditions was plotted, demonstrating its preferential binding to 3S-HS.

All proteomics data have been deposited to the ProteomeXchange Consortium via the PRIDE partner repository^25^. Annotations linking the raw data files to the experimental conditions can be found in Table 6.

**Table 6:** Interactome experiment.

Raw file name	Cohort	AE-MS bait
E24497b_PRC_9_2p_CMB-717_5_1.raw	MCI	Control
E24495b_PRC_9_2p_CMB-717_4_1.raw	MCI	Control
E24493b_PRC_9_2p_CMB-717_3_1.raw	MCI	Control
E24491b_PRC_9_2p_CMB-717_2_1.raw	MCI	Control
E24489b_PRC_9_2p_CMB-717_1_1.raw	MCI	Control
E24521_PRC_9_2p_CMB-717_5_2.raw	MCI	3S-HS 1
E24519_PRC_9_2p_CMB-717_4_2.raw	MCI	3S-HS 1
E24517_PRC_9_2p_CMB-717_3_2.raw	MCI	3S-HS 1
E24515_PRC_9_2p_CMB-717_2_2.raw	MCI	3S-HS 1
E24513_PRC_9_2p_CMB-717_1_2.raw	MCI	3S-HS 1
E24545_PRC_9_2p_CMB-717_5_3.raw	MCI	3S-HS 2
E24543_PRC_9_2p_CMB-717_4_3.raw	MCI	3S-HS 2
E24541_PRC_9_2p_CMB-717_3_3.raw	MCI	3S-HS 2
E24539_PRC_9_2p_CMB-717_2_3.raw	MCI	3S-HS 2
E24537_PRC_9_2p_CMB-717_1_3.raw	MCI	3S-HS 2
E24569_PRC_9_2p_CMB-717_5_4.raw	MCI	HS 1
E24567_PRC_9_2p_CMB-717_4_4.raw	MCI	HS 1
E24565_PRC_10_2p_CMB-717_3_4_less.raw	MCI	HS 1
E24563_PRC_9_2p_CMB-717_2_4.raw	MCI	HS 1
E24561_PRC_9_2p_CMB-717_1_4.raw	MCI	HS 1
E24593_PRC_10_2p_CMB-717_5_5.raw	MCI	HS 2
E24591_PRC_10_2p_CMB-717_4_5.raw	MCI	HS 2
E24589_PRC_9_2p_CMB-717_3_5.raw	MCI	HS 2
E24587_PRC_9_2p_CMB-717_2_5.raw	MCI	HS 2
E24585_PRC_10_2p_CMB-717_1_5.raw	MCI	HS 2
E24507_PRC_9_2p_CMB-717_10_1.raw	Control	Control
E24505_PRC_9_2p_CMB-717_9_1.raw	Control	Control
E24503b_PRC_9_2p_CMB-717_8_1.raw	Control	Control
E24501b_PRC_9_2p_CMB-717_7_1.raw	Control	Control
E24499b_PRC_9_2p_CMB-717_6_1.raw	Control	Control
E24531_PRC_9_2p_CMB-717_10_2.raw	Control	3S-HS 1
E24529_PRC_9_2p_CMB-717_9_2.raw	Control	3S-HS 1
E24527_PRC_9_2p_CMB-717_8_2.raw	Control	3S-HS 1
E24525_PRC_9_2p_CMB-717_7_2.raw	Control	3S-HS 1
E24523_PRC_9_2p_CMB-717_6_2.raw	Control	3S-HS 1
E24555_PRC_9_2p_CMB-717_10_3.raw	Control	3S-HS 2
E24553_PRC_9_2p_CMB-717_9_3.raw	Control	3S-HS 2
E24551_PRC_9_2p_CMB-717_8_3.raw	Control	3S-HS 2
E24549_PRC_9_2p_CMB-717_7_3.raw	Control	3S-HS 2
E24547_PRC_9_2p_CMB-717_6_3.raw	Control	3S-HS 2
E24579_PRC_10_2p_CMB-717_10_4.raw	Control	HS 1
E24577_PRC_10_2p_CMB-717_9_4.raw	Control	HS 1
E24575_PRC_9_2p_CMB-717_8_4_20201118231707.raw	Control	HS 1
E24573_PRC_9_2p_CMB-717_7_4.raw	Control	HS 1
E24571_PRC_9_2p_CMB-717_6_4.raw	Control	HS 1
E24603_PRC_10_2p_CMB-717_10_5.raw	Control	HS 2
E24601_PRC_10_2p_CMB-717_9_5.raw	Control	HS 2
E24599_PRC_10_2p_CMB-717_8_5.raw	Control	HS 2
E24597_PRC_10_2p_CMB-717_7_5.raw	Control	HS 2
E24595_PRC_10_2p_CMB-717_6_5.raw	Control	HS 2

Annotations of the raw data files and the corresponding cohort and AE-MS bait.

### Pull-down and Western blot experiments

The pull-down experiment was performed as described above by pooling the five different samples of the MCI or control group. Elution was performed by adding 50 μl of 10X NuPAGE Sample Reducing Agent (Life Technologies) and 4X NuPAGE LDS Sample Buffer (Life Technologies) to the beads. Elution was completed by heating the samples at 95 °C for 5 min. Protein mixtures were resolved by SDS-PAGE on a 4–12% Criterion™ XT Bis-Tris Protein Gel (Bio-Rad), loading 20 μl of each elution fraction and input samples (4 μl of each CSF replica). To assess the molecular weight, the SeeBlue™ Plus2 Pre-stained protein standard (Invitrogen) was run together with the samples. Proteins were transferred to PVDF membranes following blocking with 5% non-fat dry milk (Santa Cruz ChemCruz) in TBS with 0.2% Tween-20 (v/v) (TBS-T). Membranes were incubated at 4 °C overnight with 1:1,000 anti-antithrombin III antibody (ab126598, Abcam) in blocking solution. After 3 washes with TBS-T, membranes were incubated with 1:5,000 goat anti-rabbit HRPO-labeled antibody (32460, Invitrogen) for 1 h at room temperature. After 3 washes with TBS-T, the membranes were incubated with SuperSignal™ West Dura Extended Duration Substrate (Thermo Scientific) for 5 min and the chemiluminescent signal was captured using an Amersham Imager 600 (GE Healthcare). Figure 4 depicts the results of the Western blots for ATIII in the MCI (Fig. 4c) and control (Fig. 4d) groups. Full uncropped western blot results are in the Figshare Repository (Uncropped WB^23^).

## Data Record

For each sample group (MCI and control), 5 biological replicates (CSF from 5 different individuals) were used. All proteomics data have been deposited to the ProteomeXchange Consortium via the PRIDE partner repository^25^. The annotations of the raw data files to the experiment conditions can be found here:

Processed data are available in the Figshare Repository^23^, including:

- A word file with all the proteins lists generated during the processing (Proteins list)
  - The list of proteins selected as potential interacting partners of the baits (Table 1–8)
  - The list of proteins that bind preferentially to 3S-HS 1 in MCI sample group (Table 9)
  - The list of proteins that bind preferentially to 3S-HS 2 in MCI sample group (Table 10)
  - The list of proteins that bind preferentially to 3S-HS 1 in control sample groups (Table 11)
  - The list of proteins that bind preferentially to 3S-HS 2 in control sample group (Table 12)
  - The list of unique proteins in the MCI CSF proteome (Table 13)
  - The list of unique proteins in the control CSF proteome (Table 14)
  - The list of proteins shared between MCI and control CSF proteomes (Table 15)
- Two PDF files containing the correlation plots of all identified proteins between the different replicas for the MCI and control sample groups (Correlation plot)
- A PDF file with the uncropped western blot membranes for ATIII protein (Uncropped WB)

## Technical Validation

The data were generated using CSF samples of ten human individuals; five with diagnosed MCI and five age-matched controls. For our analysis, for each sample group (MCI and control), the five samples were considered biological replicas. Two types of analysis were performed: a global CSF proteome profiling and 3-*O* sulfated HS interactome profiling. For the latter, for each sample, five different baits were used, being (1) a control bait (beads-only), (2) 3-*O* sulfated HS 1, (3) 3-*O* sulfated HS 2, (4) HS 1 and (5) HS 2. Baits 4 and 5 are HS that have the same structure as baits 2 and 3, respectively, but without any 3-*O* sulfation. The full list of proteins quantified in the affinity-enrichment assay, with the corresponding Log_2_ fold change, p-value, FDR and number of proteins where they were identified can be found in the Figshare Repository (Proteins List^23^).

Proteome profiling of the different input samples was performed to assess how different the input samples were and if any such differences could help to explain any potential differences in the interactomes from the MCI and control sample groups we would later on obtain. Here, 759 proteins were identified and, following the selection of proteins with a minimum of 3 valid LFQ values in at least one condition, 434 proteins were quantified. Assessment of the data demonstrated high correlation values (R^2^ > 0.9) of the identified proteins between replicas (Fig. 2a left panel) and an overall clustering of the replicas in the PCA plot (Fig. 2a right panel). This indicates that the replicas of each sample group show overall a high concordance. To compare the proteome of the MCI and control sample group, proteins present in less than 3 replicas of each group were excluded. This method allowed the quantification of 74 proteins unique to the MCI samples, 5 proteins unique to the control samples, and 355 proteins shared between both groups. A number of these 74 proteins (YWHAG, TREM2, GALNT2, CLSTN3, NECTIN 3, PGK1, and others) found to be unique to the MCI CSF proteome are shared with previous similar studies^30–32^. However, we did not identify other well-described CSF biomarkers such as APP or MAPT. This may be explained by different stages of AD (MCI vs (asymptomatic) AD; CDR scores > 1) analyzed in other studies. Also, the mode of data acquisition (DDA vs DIA) which, in the case of this study, can bias the identification to the most abundant proteins in the sample and, thus, mask the presence of lower abundant proteins. Finally, the type of assay (TMT-labeled vs label-free) and the LC-MS/MS equipment sensitivity can contribute to discrepancies between our results and other reports. Nevertheless, to select proteins significantly (FDR < 0.05 and log_2_ FC > 1) different between the sample groups, statistical testing comparing the means of the LFQ values between MCI and controls was performed using the limma package. According to this analysis, no proteins showed differential regulation between the groups. This might indicate that the unique proteins identified in the MCI and control proteomes have a low abundance and hence, did not show statistically significant expression.

In the AE-MS studies, 1,051 proteins were identified, and 698 proteins could be quantified. Assessment of the data demonstrated rather low correlation values (R^2^ < 0.9) of identified proteins between some of the replicas and poor clustering of the replicas in the PCA plots. This seems to indicate that the AE samples have an extra level of variation compared to the input samples, which was taken into account during the statistical analyses.

The interactomes of the four different HS were profiled by excluding proteins equally present in the AE beads-only (control) condition. With this analysis, 24, 17, 14, and 6 proteins were identified as interactors of the 3S-HS 1, 3S-HS 2, HS 1, and HS 2 conditions in the MCI sample group respectively. In the control sample groups, 22, 38, 20, and 6 proteins were selected as interactors of 3S-HS 1, 3S-HS 2, HS 1, and HS 2 conditions, respectively. UpSet plot (Fig. 3a,b) was used to observe the intersection of the different interactome and allowed the distinction of proteins that bind HS, regardless of the presence of 3-*O* sulfation. Of those, LAMB1, RARRES2 and CAL3A1 have not yet been reported to interact with HS. However, SOD3^33^ and SPON1^34^ are known HSPGs-interacting proteins, providing support to our dataset.

To identify proteins with preferential binding to 3S-HS, the 3S-HS 1/3S-HS 2 conditions were compared to HS 1 and HS 2 conditions, and proteins with significantly increased LFQ values detected in at least 3 of the 5 replicas were selected as interactors that bind preferably to 3-*O* sulfated HS. With this analysis, 6 and 11 proteins were selected in the 3S-HS 1 and 3S-HS 2 conditions of the MCI group respectively (Fig. 3c), and 10 and 27 proteins were selected in 3S-HS 1 and 3S-HS 2 conditions of the controls group respectively (Fig. 3d).

Using an AE-MS approach to profile the 3S-HS interactome in the human CSF, we identified ATIII, a known 3S-HS interactor. FGFR3 was also identified as a potential 3S-HS interactor similar to previous reports demonstrating the interaction between (3S-)HSPGs and the FGF(R) family. Other (3S)HS-interacting proteins were also newly identified, such as TMEM132E, calsyntenin-1, and LRP1. Strikingly, LRP1 was previously reported to interact with HSPGs^35,36^, although the nature of such interaction and the structural requirements are not well understood. Here we demonstrated, for the first time, using the above-mentioned approach we are able to identify HS interaction dependent and independent of 3-O sulfation. The fact that some proteins identified are in line with previous reports, demonstrates the reliability of the dataset generated.

The profile plot of ATIII (Fig. 4a) demonstrates the binding preference of the protein to 3S-HS 2. We validated the 3S-HS interaction of ATIII detected via LC-MS/MS using pull-down experiments (Fig. 4c,d). The ATIII protein is only present in the eluted fractions of the pull-downs performed with the 3S-HS 2 oligosaccharide, confirming the known literature that ATIII binds to HS containing 3-O sulfation flanked by 2-*O* sulfated iduronic and glucuronic acid moieties. Of note, no other bands were observed in the Western blot (Uncropped WB^23^). This validation provides confidence and strength to the interactome dataset profiled in this study.

## Data Availability

No custom code was used to generate or process the data presented in this manuscript. All software used in this work is in the public domain, with parameters being clearly described in Methods. If no detailed parameters were mentioned for a software, default parameters were used as suggested by the developer.
